# Triple Synchronous Primary Melanomas in a 77-Year-Old Sea Captain: Importance of Total Skin Examination

**DOI:** 10.7759/cureus.33511

**Published:** 2023-01-08

**Authors:** Aleksandra Nowicka, Christopher Rowland Payne

**Affiliations:** 1 Dermatology, Nicolaus Copernicus University, Bydgoszcz, POL; 2 Dermatology, Private Practice, London, GBR

**Keywords:** multiple melanomas, complete skin examination, multiple primary melanomas, synchronous melanomas, synchronicity, dupuytren’s contracture, diagonal ear lobe creases, index lesion focused examination, total skin examination, synchronous primary melanomas

## Abstract

When it comes to skin diseases, melanomas are considered the most lethal. Triple synchronous primary melanomas (SPMs) are rare. Here we have reported a case of a 77-year-old white male with three SPMs. The patient presented with a nodular melanoma in the upper left back; total skin examination (TSE) revealed additional melanomas in the right retroauricular region and on the left arm. The patient was unaware of these two additional melanomas that were only found because of the routine TSE, and the index lesion focused examination would have missed both. We advocate that TSE should be practised routinely by all dermatologists for all new patients, and also, from time to time in patients attending long-term follow-up appointments. Early diagnosis means simpler treatments and more favourable outcomes.

## Introduction

Melanoma, the most lethal of skin diseases, is responsible for 70% of all skin cancer deaths in the UK [1]. Melanoma is the sixth most common cancer in men and becomes more common as people age [1-3]. Each year, there are around 2300 melanoma deaths in the UK, and melanoma incidence rates have increased in males by 38% over the last decade [1]. Here we present the case of a 77-year-old man who had developed three synchronous primary melanomas (SPMs). This rare case highlights the importance of routine total skin examination (TSE) in SPM diagnosis.

## Case presentation

**Figure 1:**
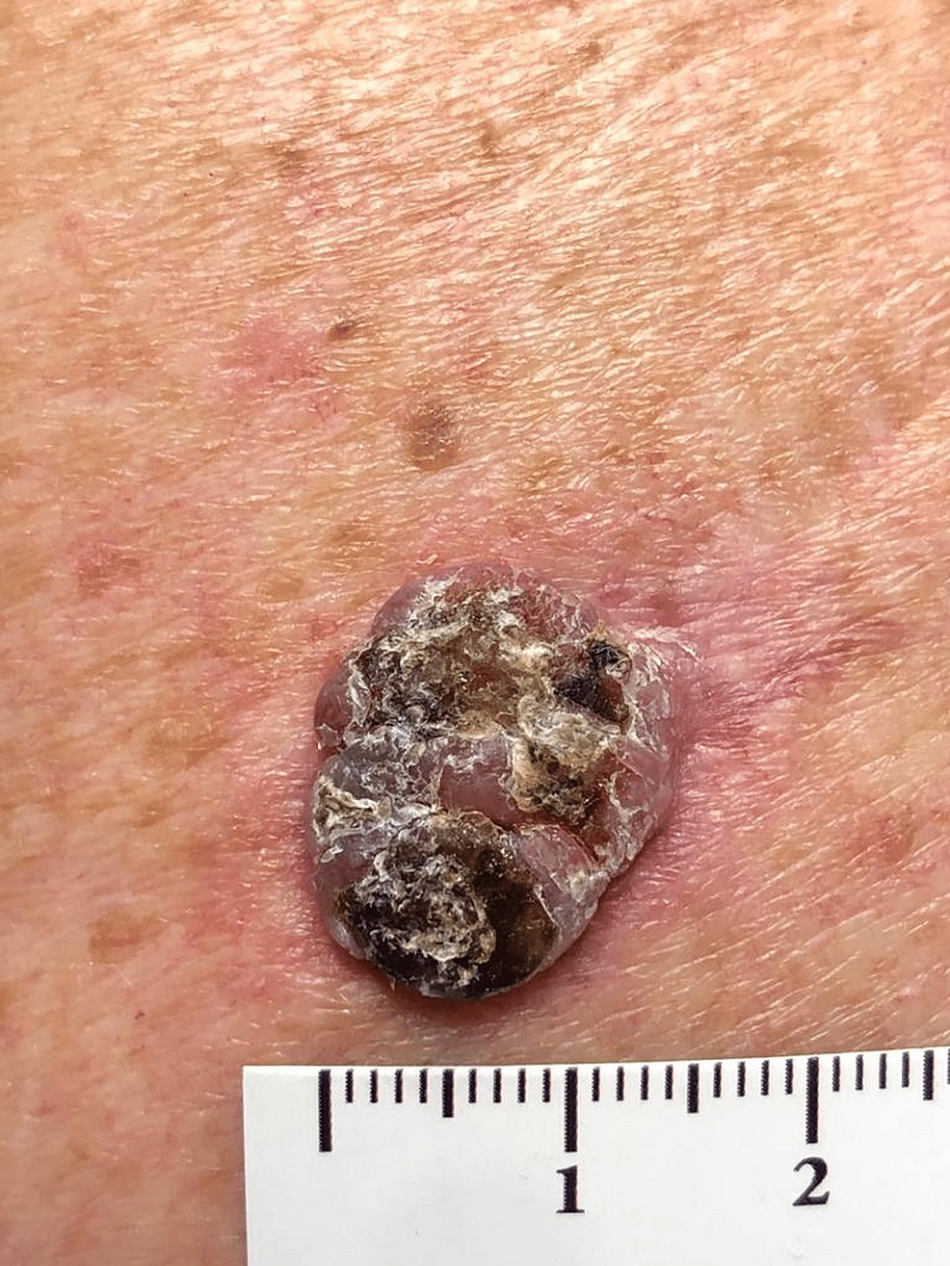
Nodular melanoma of the upper left back It was ulcerated, with a diameter of at least 2 mm, Breslow thickness at least 4.9 mm, Clark level at least IV, mitotic rate 7 per mm^2^, and microstaging pT4b.

**Figure 2:**
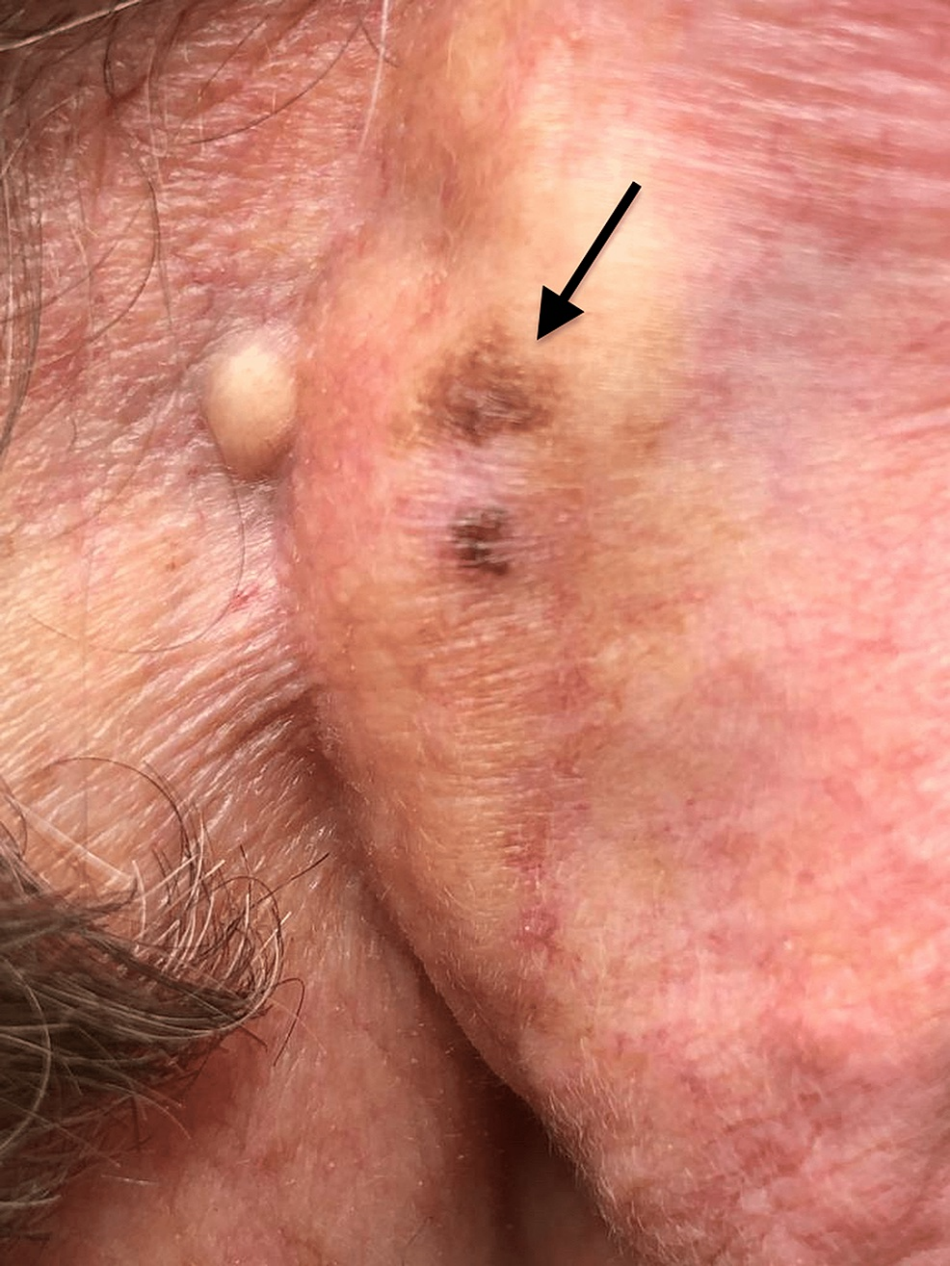
Lentigo maligna/early invasive melanoma of the right retroauricular region It had a Breslow thickness of 0.2 mm; the arrow points to the melanoma.

**Figure 3:**
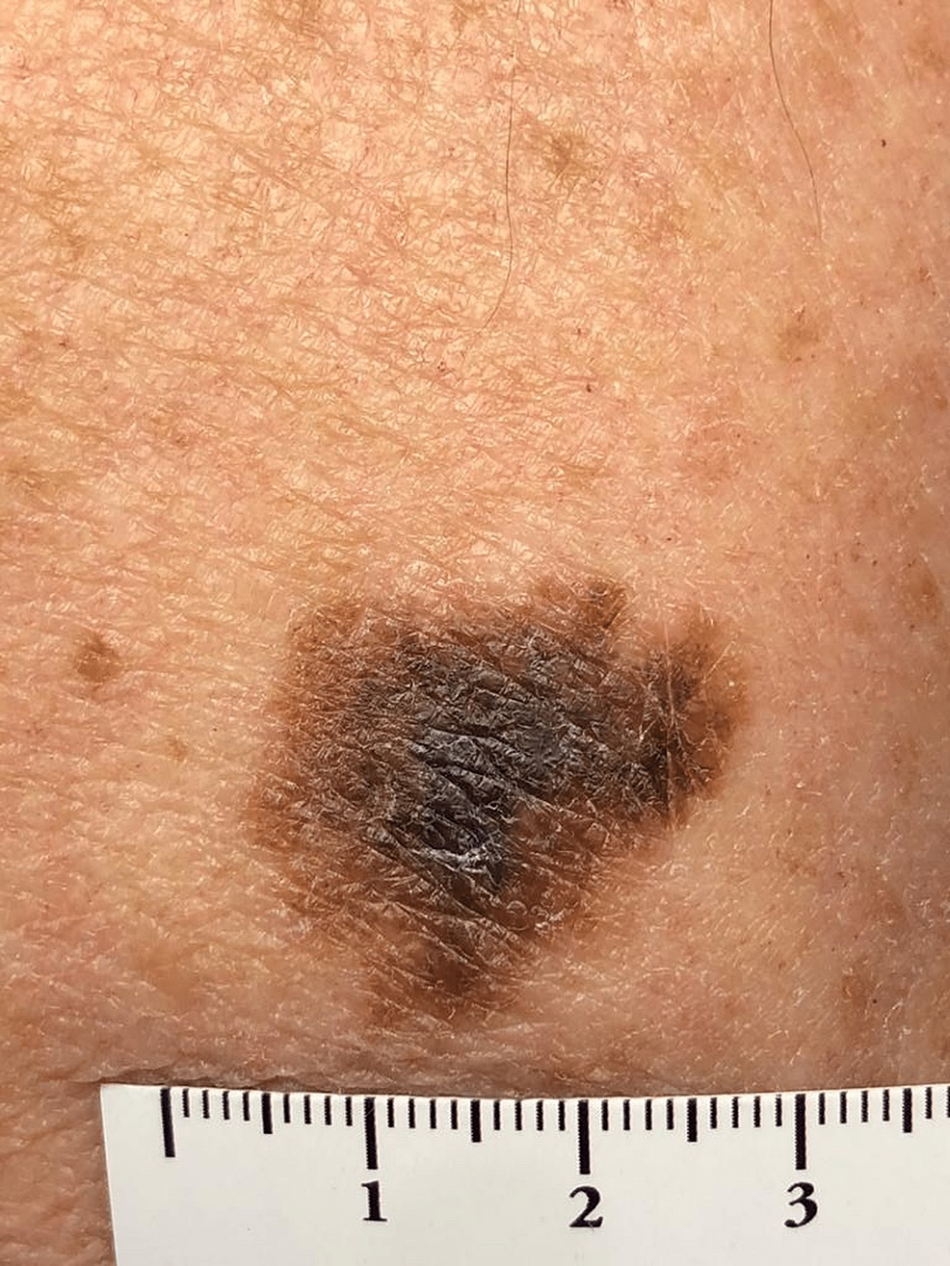
Melanoma of the left arm, in situ

A 77-year-old male sea captain (skin phototype II), with nicotine addiction, hyperlipidemia and prostate cancer, had a lifelong history of sun exposure. He was referred to the dermatology department with a one-month history of a bleeding exophytic lesion on his upper left back (Figure 1). TSE revealed two further highly suspicious pigmented lesions in the right retroauricular region (Figure 2) and on the left arm (Figure 3). There was no lymphadenopathy. All three lesions were microscopically confirmed to be three separate primary melanomas: the upper back lesion was a nodular melanoma (ulcerated, with a diameter of at least 2 mm, Breslow thickness at least 4.9 mm, Clark level at least IV, mitotic rate 7 per mm^2^; microstaging pT4b), the retroauricular lesion was a lentigo maligna/early invasive melanoma (Breslow thickness 0.2 mm) and the arm lesion was a melanoma in situ. Definitive excision was undertaken for all three lesions. The fluorodeoxyglucose (FDG)-positron emission tomography (PET) scan and sentinel node biopsy were negative. Physical examination revealed solar keratoses and bilateral Dupuytren’s contracture (DC). TSE also revealed diagonal earlobe creases (DELC), which is a surrogate marker for cardiovascular diseases. It led to a new diagnosis of atrial fibrillation and initiation of anti-arrhythmic and anti-coagulation therapies. Following surgery, despite several written and telephone reminders, the patient was lost to follow-up.

## Discussion

Synchronicity is defined as two or more events occurring at the same time [4]. Strictly speaking, identifying second melanomas at a follow-up consultation cannot be described as synchronous and indeed suggests that the patient was insufficiently examined at the initial consultation. Such cases should not be considered as SPMs.

A thorough clinical examination, regardless of the reason for a consultation, is crucial in discovering any lesions that are not the presenting complaint. TSE conducted by a dermatologist not infrequently reveals other coincidental cutaneous malignancies and sometimes other cutaneous or general medical physical signs of clinical importance. Both index lesion focused examination (ILFE) and examination of only the sun-exposed skin put a patient’s life at risk. In a case series by Rowland Payne, of 104 melanomas, 30 were discovered on routine TSE [5]. In another case series by Aldridge et al., from a pigmented lesion clinic, one third of melanomas detected were not the lesions for which the patients had been referred for the assessment of possible melanomas, i.e., one third of the melanomas detected were an incidental diagnosis made only because of TSE [6]. Thus, examination only of the referred index lesions would have missed one third of melanomas in both these studies. In a third case series, 56% of melanomas detected in a general dermatology practice were found as a result of a dermatologist-initiated TSE, and not as a result of patient complaints [7]. As yet, neither randomised controlled trials nor meta-analyses exist comparing the benefits of TSE with those of ILFE.

Good prognosis of malignant lesions depends upon early diagnosis [2,3,8]. Early diagnosis means simpler, less costly treatment and more favourable outcomes. Melanomas diagnosed incidentally on TSE are thinner and more likely to be in situ [2,6-8]. Since survival is related to the melanoma lesion thickness, it is prudent to stress the importance of TSE [2,8]. Performing TSE routinely, irrespective of the presenting cutaneous complaint, makes an important difference to the total number of skin malignancies diagnosed early [9,10]. For example, in a study of skin malignancies occurring in patients with DC, 24% of skin malignancies were reported by patients whereas 55% were discovered on TSE (Paper: Bogdanov I, Rowland Payne C. Total Skin Examination Discovers Skin Cancers Early in Patients With Dupuytren’s Contracture. 24th European Academy of Dermatology & Venereology Congress; October 7-11, 2015).

The benefits of TSE are not limited to early diagnosis of incidental melanomas. TSE often reveals other important diagnoses or physical signs. In a prospective series of 200 consecutive general dermatology consultations, 41 patients had at least 117 skin malignancies, up to that moment unrecognised either by the patient or the referring doctor (including six patients with melanomas and one with cutaneous T-cell lymphoma), as well as one patient with prostate cancer, one with an aggressive desmoid tumour and three patients with myelodysplasia (preleukemia), 11 with autoimmune disease (including one with systemic lupus erythematosus and two with systemic sclerosis) and 39 with psychological disorders (most often anxiety or depression) [11]. During TSE, our patient was noted to have Frank's sign of diagonal ear lobe creases, a sign of vascular disease, which led to a new diagnosis of atrial fibrillation [12]. In this case, TSE became a treasured tool to discover underlying heart disease that, together with the patient’s long history of smoking and hyperlipidemia, was putting him at an enhanced risk of stroke. Because of TSE revealing DELC, preventative anti-thromboembolic treatment was initiated in our patient.

Noteworthy is that TSE revealed early bilateral DC in our patient. DC patients were found to have an increased risk of skin malignancies, occurring in 30% cases, and were also found to have a 24% increase in the overall relative risk of internal malignancies [13,14]. Indeed our patient had prostate cancer. Regular general medical follow-ups as well as TSE is recommended in DC patients as DC is associated with an enhanced risk of not only skin and internal malignancies but also cardiovascular diseases [13,15].

It can be argued that TSE should be adopted by all dermatologists as a routine for both new patients and patients coming for long-term follow-up appointments. Even though the majority of patients have a preference to undergo TSE, some may feel embarrassed and uncomfortable as it requires them to be fully undressed [16]. An understanding and professional approach by the doctor is crucial and allows TSE to be conducted correctly and respectfully, and in most cases, with the assistance of a chaperone. Due to time constraints imposed by the UK National Health Service (NHS), doctors may feel pressured to practice ILFE rather than TSE because they have limited time to treat each patient. Nevertheless, the professional responsibility of the doctor is to the patient in front of him or her and not to the system in which they work. Examining each patient properly consumes some extra time and resources now, but early diagnosis of melanoma clearly saves time and resources later.

Of particular interest are the medical voices against the sometimes life-saving procedure of TSE. Many physicians consider that this non-invasive and rapidly performed procedure is time consuming. This is perplexing as the median time spent on TSE in one study was only 70 seconds [17]. Indeed, it could be argued that TSE is a moral obligation incumbent upon dermatologists, i.e., part of the dermatologist’s professional duty to the patient.

## Conclusions

To conclude, TSE revealed clinically useful and potentially life-saving information in this 77-year-old male patient, who had been completely unaware of both his multiple primary melanomas and atrial fibrillation. We believe that TSE should be offered by all dermatologists during the examination of both routine, new patients and long-term follow-up patients, as ILFE misses much.
